# An Achiasmatic Mechanism That Ensures the Regular Segregation of Sex Chromosomes in Male Meiosis in the Black Spongilla-fly *Sisyra nigra* (Retzius 1738), Sisyridae, Differs from the Mechanism Commonly Observed Within Neuroptera

**DOI:** 10.3390/insects16121273

**Published:** 2025-12-15

**Authors:** Seppo Nokkala, Christina Nokkala

**Affiliations:** Laboratory of Genetics, Department of Biology, University of Turku, FI-20014 Turku, Finland; chrinok@utu.fi

**Keywords:** *Sisyra nigra*, sex chromosomes, achiasmatic segregation mechanisms, touch-and-go pairing, distance pairing

## Abstract

The family Sisyridae, the Spongilla-flies, is notable for its phylogenetic position as a basal group within Neuroptera. Using the improved Schiff-Giemsa method, we analyzed the behavior of the sex chromosomes X and Y during male meiosis in *Sisyra nigra* (Retzius 1738). The diploid chromosome number in males was 2*n* = 12 + XY. In pachytene, X and Y chromosomes appeared positively heteropycnotic and loosely paired. In early diakinetic nuclei, autosomal bivalents typically exhibited one distally located chiasma, although bivalents with two chiasmata were occasionally observed. The X and Y univalents were isopycnotic with the autosomes, with the X considerably larger than the Y. During the first meiotic division, metaphase plates were radial, with autosomal bivalents forming a ring and X and Y univalents positioned centrally, well separated from each other. In metaphase cells, X and Y were located at the equator, strongly indicating their amphitelic orientation. However, they later formed a pseudobivalent from which X and Y segregated simultaneously with autosomal half bivalents at anaphase I. This achiasmatic segregation mechanism, touch-and-go pairing, has now been observed for the first time in a species carrying chromosomes with a localized centromere. At the second metaphase, two cell types were observed: one with the X chromosome and the other with the Y chromosome. The behavior of the sex chromosomes in *S. nigra* is notably different from that in other Neuroptera, where sex chromosomes exhibit syntelic orientation and distance pairing at metaphase I. The unusual mechanism of sex chromosome segregation in the family Sisyridae aligns well with molecular phylogenetic findings concerning the family’s basal position within the order Neuroptera.

## Simple Summary

This chromosomal study examines the meiotic behavior of the sex chromosomes X and Y during male meiosis in the black Spongilla-fly *Sisyra nigra* (Retzius 1738), family Sisyridae, in the insect order Neuroptera. It was observed that the X and Y chromosomes remain at the first division metaphase plate in the center of a ring formed by autosomal bivalents until late metaphase. The sister centromeres of the sex chromosomes are connected to opposite spindle poles with spindle fibers. The sex chromosomes form a chromosome pair just before the onset of the first anaphase, in which they separate to opposite spindle poles. A mechanism of this kind, known as touch-and-go pairing, is described for the first time in Neuroptera. In Neuroptera, the sex chromosomes usually separate via a process called distance pairing. The name reflects the situation at the first metaphase. Both sister centromeres of the sex chromosomes are attached to the same spindle pole by spindle fibers. However, the X and Y chromosomes are connected to opposite poles, and they are positioned near the poles in the metaphase spindle. The findings support molecular studies about the relationships within Neuroptera and highlight the need for further chromosomal research.

## 1. Introduction

Neuroptera (lacewings and antlions) is the largest, most species-rich, and most morphologically diverse of the three orders, Megaloptera, Raphidioptera, and Neuroptera, which together make up the superorder Neuropterida. Withycombe [1] already outlined the basic principles of Neuropteran phylogeny, and the idea of monophyly of the order has persisted, primarily based on larval morphology and anatomy. Contemporary molecular phylogenies, based on mitogenome and transcriptome analyses, align well with earlier morphology-based phylogenies [2,3,4,5,6].

Within Neuroptera, Coniopterygidae (dustywings) are considered distinct from all other members of the Neuroptera, and it is the most basal family in the order, not included in Euneuroptera [7]. Sisyridae (Spongilla-flies), along with Nevrorthidae (dragon lacewings), are grouped with Osmylidae (lance lacewings) within the superfamily Osmyloidea. The superfamily Osmyloidea is monophyletic and forms a basal sister clade to all remaining Neuroptera. The family Sisyridae has fossil records dating back to the mid-Cretaceous [8]. Today, it is a relatively small insect family, with about 70 extant species across four genera worldwide [7]. In Finland, three species are present, with only *Sisyra nigra* being common and found throughout the country [9].

Cytogenetic information on the basal groups of Neuroptera is limited. There are no existing records regarding the cytogenetic characteristics or even chromosome numbers for Conioterygidae, Osmylidae, or Nevrorthidae. The only available chromosomal data come from species in the family Sisyridae, and even this data is sparse. Hughes-Schrader [10] studied male meiosis in *Climachia areolaris* (Hagen). She found the chromosome number to be *n* = 6 + X0. Additionally, she described the X chromosome as being positioned in the metaphase I spindle near one of the spindle poles. She also observed that in *Sisyra vigaria* (Walker), the chromosome number was *n* = 6 + XY. The X and Y chromosomes appeared as a bivalent or as univalents in metaphase I cells. She claimed that the univalent X and Y chromosomes had a syntelic orientation, meaning they were attached to opposite spindle poles and, as a result, displayed distance segregation. Her findings were illustrated with drawings. However, the methods used were quite outdated even at that time and poorly suited for this study, leaving these observations open to alternative interpretations. This behavioral pattern of sex chromosomes, so-called distance pairing, was first described in antlions (Mantispidae) by Naville and de Beaumont [11]. The phenomenon was later observed in several other neuropteran families, including Chrysopidae, Mantispidae, Sisyridae, Osmylidae, and Hemerobiidae [10,12,13,14,15], and was suggested to apply to the Neuroptera in general [16].

Currently, the behavior of sex chromosomes in neuropteran male meiosis is well understood in groups where it has been studied [11,15,16,17]. The sex chromosomes are loosely paired at pachytene and appear as univalents in diakinesis. During prometaphase, the univalents, along with the autosomal bivalents, are brought to the equatorial plane. During late prometaphase, the bivalents attain their proper bipolar orientation, and syntelically oriented X and Y univalents are pulled near opposite spindle poles. This arrangement is known as distance pairing. The segregation mechanism is quite accurate. Only 0.6% nondisjunction of the X and Y has been recorded [15].

Currently, chromosome-level genome assemblies have become a new source of karyotypic information for species. Using this method, seven pseudochromosomes, including the X chromosome, were assembled for two representatives of the neuropterid family *Sisyra*, *S. nigra* [18] and *S. terminalis* [19].

In the present study, we investigated the black Spongilla-fly *Sisyra nigra* for two reasons. Firstly, the family Sisyridae appears in a basal position within the main body of Neuroptera, excluding the Coniopterygidae. We aimed to determine whether it is possible to identify chromosomal characteristics that support the basal position of the Sisyridae in morphology-based and molecular phylogenies, with a particular focus on the behavior of the X and Y chromosome univalents during male meiosis. Secondly, we wanted to determine the relationship between pseudochromosomes and actual physical chromosomes. To achieve these objectives, we employed improved cytological methods, including hypotonic treatment of the material prior to fixation and slide preparation in 45% acetic acid, followed by staining of air-dried slides. This approach greatly enhanced the documentation of the results through photography.

## 2. Materials and Methods

### 2.1. Insects

Eleven males were collected in Forssa, Matku, Finland (60°39′20.6″ N, 21°19′5.4″ E) between 11 July and 3 August 2025, from plants by a small lake, and two males from Kiparluoto, Kustavi, Finland (60°58′36.1″ N, 23°19′5.4″ E) on 1 September 2025, from plants by the Baltic Sea. Testes were dissected in a hypotonic 0.8% tri-sodium citrate (Riedel-de Haën, Sleeze, Germany) solution and allowed to stand there for 8–9 min, then transferred to ethanol (Anora Group Oyj, Rajamäki, Finland)–acetic acid (3:1) (Honeywell/Fluka, Sleeze, Germany) fixative and stored at +4 °C until slide preparation.

### 2.2. Slide Preparation

Air-dried preparations were made by macerating testicular follicles in a drop of 45% acetic acid on a glass microscope slide, then gently squashing them under a coverslip. The slides were examined under a phase-contrast microscope to assess the presence and quality of meiotic stages. Selected slides were collected on dry ice. Cover slips were removed, and the preparations were dehydrated in freshly prepared 3:1 fixative for 45 min and air-dried. The air-dried slides were stored in a dust-free place.

### 2.3. Slide Staining

Air-dried slides were stained using the Schiff-Giemsa method, as described by Grozeva and Nokkala [20]. In brief, slides were incubated in 1 N HCl (Honeywell, Fluka) at room temperature for 20 min, hydrolyzed in 1 N HCl at 60 °C for 6 min, stained with Schiff’s reagent for 15 min, thoroughly rinsed in distilled water, incubated in Sorensen’s phosphate buffer (pH 6.8) for 5 min, and stained with 4% Giemsa (Sigma-Aldrich, Merck KGaA, Darmstadt, Germany) in Sorensen’s phosphate buffer (pH 6.8) for 20 min. After a brief rinse with distilled water, the slides were air-dried for 20 min and mounted in Entellan™New (Sigma-Aldrich, Merck KGaA) mounting medium.

### 2.4. Documentation

Chromosome preparations were photographed using a Nikon DS-Fi3 camera (Tokyo, Japan) mounted on a Nikon C1-L microscope (Tokyo, Japan), with NIS-Elements software (version 5.01.00). Final processing of photomicrographs was performed using Corel PhotoPaint 2025.

## 3. Results

Although the material was collected over nearly two months, the distribution of meiotic stages in the testes remained remarkably consistent. The longer-lasting stages, metaphase I and pro-metaphase I, were common, whereas anaphases were rarely observed.

**Figure 1:**
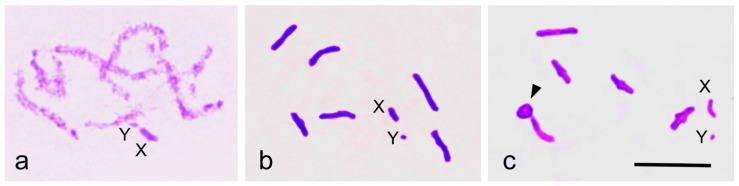
(**a**–**c**) Prophase of the first meiotic division in *S. nigra* male. (**a**) Pachytene, positively heteropycnotic sex chromosomes are loosely paired. (**b**) Diakinesis, six autosomal bivalents and X and Y univalents. (**c**) Diakinesis, one of the autosomal bivalents has two chiasmata (ring bivalent, arrowhead), and X and Y are univalents. Bar equals 10 µm.

During pachytene, the X and Y sex chromosomes were clearly positively heteropycnotic and appeared as a loosely paired structure (Figure 1a). At diakinesis, six autosomal bivalents of approximately equal size, along with univalent X and Y chromosomes—where the X chromosome is notably larger than the Y—are visible (Figure 1b). The chromosome composition is thus 2*n* = 12 + XY. Early diakinesis commonly reveals one chiasma near or at the telomere on most bivalents (Figure 1b). Occasionally, one or rarely two bivalents display two (sub-)terminal chiasmata, forming ring bivalents (Figure 1c).

**Figure 2:**
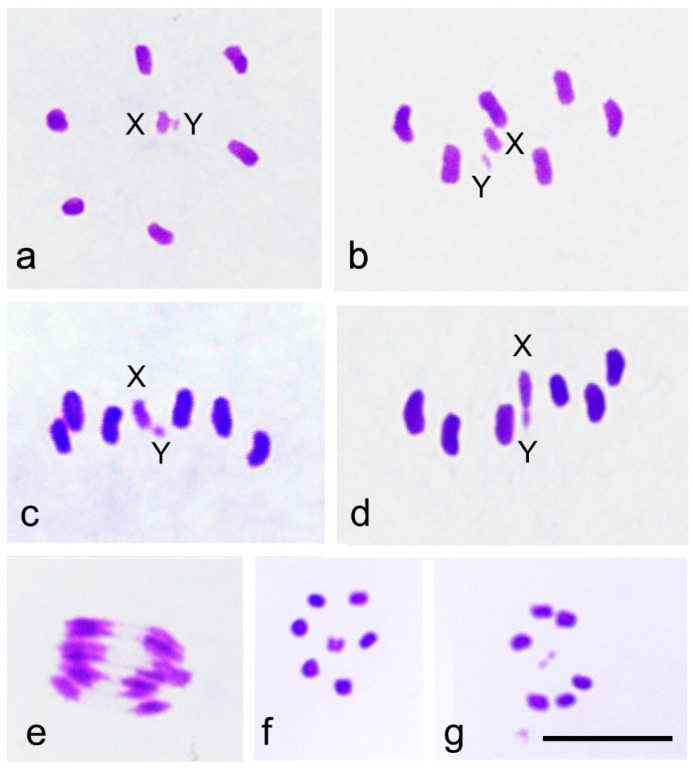
(**a**–**g**) Meiotic divisions in *S. nigra* male. (**a**) Metaphase I in polar view. Autosomal bivalents form a ring with the physically unconnected X and Y chromosomes located at the center. (**b**) Metaphase I, viewed from a slightly oblique perspective above the equatorial plane, shows the X and Y chromosomes clearly positioned at the equator. (**c**) Metaphase I in side view. The sex chromosomes lie at the equatorial plane. (**d**) Late metaphase I with the XY pseudobivalent. (**e**) Anaphase I. Sex chromosomes move simultaneously with autosomal half-bivalents towards the poles. (**f**) Metaphase II with the X chromosome. (**g**) Metaphase II with the Y chromosome. Bar equals 10 µm.

Examination of approximately 400 metaphase I plates revealed a radial metaphase configuration and a consistent placement of the sex chromosomes in the center of the ring formed by autosomal bivalents (Figure 2a). The consistent equatorial localization of the X and Y suggests their amphitelic orientation (Figure 2b,c). Not a single cell was found where the univalents were above or below the equatorial plane near the poles, indicating syntelic orientation. However, despite their amphitelic orientation, the X and Y do not divide during the first meiotic division but instead form a pseudobivalent just before the onset of anaphase I (Figure 2d,e). This causes them to segregate from each other, resulting in two types of metaphase II plates: one containing the X chromosome (Figure 2f) and the other containing the Y chromosome (Figure 2g). Metaphase II is radial, with the single sex chromosome in the center of the ring formed by the autosomes. This mechanism, known as the touch-and-go segregation of the first meiotic division, ensures the proper segregation of the X and Y chromosomes during the first division in *S. nigra*. Sex chromosomes divide during the second division along with the autosomes.

## 4. Discussion

In insects, the sex chromosomes, X and Y, typically appear as univalents in male meiosis, and an achiasmatic segregation mechanism ensures their regular separation. Several mechanisms operate at different stages of meiosis, providing evolutionary opportunities often overlooked in meiotic research. In this study, we have observed that in *S. nigra* the univalents stay at the equatorial plane at metaphase I, indicating their amphitelic orientation, and form a pseudobivalent right at the onset of anaphase I, meaning they display touch-and-go pairing, which ensures the segregation of X and Y chromosomes during the first meiotic division. Despite their amphitelic orientation, the X and Y chromosomes are unable to divide in the first meiotic division. The touch-and-go pairing mechanism is identified for the first time in a species with chromosomes that have a localized centromere. Wilson [21] already described and named this type of pseudobivalent formation as the touch-and-go process.

Earlier, Hughes-Schrader [10] examined male meiosis in *Sisyra vicaria* (Walker). She described two types of first metaphases, one with X and Y lying separate in the center of the plate, showing, in her opinion, syntelic orientation and distance segregation, and the other with the X and Y forming a bivalent. She could not understand the connection between these two arrangements. As a matter of fact, we have just explained the very same situation in *S. nigra*. It is evident that Hughes-Schrader [10] has misinterpreted her observations and not realized that the univalency of the X and Y precedes brief pseudobivalent formation before anaphase I begins. In Figure 14 in [10], the sex chromosomes are not positioned near opposite poles but at the equator.

Details of the touch-and-go pairing mechanism have been examined in the bug species *Coreus marginatus* (L.) (Coreidae, Hemiptera), which carries holocentric chromosomes [22]. In this study, a staining method was employed that enabled the simultaneous staining of both chromosomes and centrioles. Centrioles reveal the centrosomes that form the spindle and provide a way to visualize the orientations of cells on a slide. The karyotype of this species includes a small m-chromosome pair. In some primary spermatocytes, m-chromosomes appear as a bivalent. However, in most spermatocytes, m-chromosomes appear as univalents. Univalents are negatively heteropycnotic from diplotene onwards. In early prometaphase, m-chromosomes are located near opposite asters. From there, they congress together with autosomal bivalents towards the equatorial plane, forming a ring. Then, the stabilization of metaphase occurs, during which the m-chromosome univalents move along the equatorial plane towards the center of the radial metaphase plate, where they form a pseudobivalent. When anaphase I begins, the m-chromosomes move to opposite poles simultaneously with the autosomal half-bivalents. The movements of m-chromosomes along the equatorial plate demonstrate their connections to both poles via spindle fibers, which is equivalent to the amphitelic orientation of monocentric chromosomes.

In the lace bug family Tingidae, Heteroptera, the sex chromosomes X and Y appear as univalents during male meiotic prophase and form a pseudobivalent at the center of a radial metaphase I. Pseudobivalent formation leads to the pre-reductional segregation of X and Y in this family [23]. Prereductional behavior of sex chromosomes is unusual in Heteroptera, since typically in this insect order, sex chromosomes appear as univalents at metaphase I and undergo post-reductional meiosis, that is, they divide in the first meiotic division. Their daughter chromosomes form a pseudopair at the second metaphase and separate at the second anaphase, showing touch-and-go pairing of the second meiotic division [24]. To our knowledge, touch-and-go pairing of the second division has not been observed in any other group.

It is important to note that in a species with holocentric chromosomes, the formation of the pseudobivalent is completed immediately when the metaphase plate forms, as described in *C. marginatus* above [22]. In contrast, in *S. nigra*, which has chromosomes with localized centromeres, sex chromosome univalents remain side by side on the metaphase plate for a more extended period, and the XY pseudobivalent forms just before the onset of anaphase I. The late metaphase I plates can be reliably identified because all metaphases in a cyst include a pseudobivalent.

It is unlikely, however, that the touch-and-go pairing mechanism present in *S. nigra* will be found throughout the family Sisyridae. Hughes-Schrader described a clear case of distance pairing from a population of the sisyrid *Climacia areolaris* [10]. She states that the univalent X chromosome has a syntelic orientation in metaphase I cells. Distance pairing is an achiasmatic segregation mechanism observed in most families within Neuroptera, ensuring the regular segregation of X and Y univalents. This mechanism is also reported in Coleoptera, at least in *Zopherus haldemani* and *Hyperaspis billoti*, as well as in *Phenrica austriaca* and *Disonycha glabrata*, which have multiple Y chromosomes (for a review, see [25]).

The distance pairing mechanism has been examined in detail in males of *Hemerobius marginatus* Stephens, 1836 (Neuroptera) [15,26]. Segregating univalent sex chromosomes, X and Y, are positively heteropycnotic and loosely paired in pachytene cells. Only at this stage do they show physical contact during meiosis. Hughes-Schrader had already noted in 1969 that in Mantispidae, the X and Y do not form a bivalent at any stage of meiosis [14]. In *Hemerobius*, they appear as univalents in diakinesis and congress well separated to the equatorial plane at late prometaphase. During the stabilization phase of metaphase structure, bivalents reach their proper bipolar orientation, and syntelically oriented sex chromosome univalents move near opposite poles of the metaphase I spindle. The univalents are located inside the spindle and do not reorient during the first metaphase [15].

In addition to the usual segregation of sex chromosomes, distance pairing can also enable preferential segregation of B chromosome univalents within the same cells. However, these B chromosome univalents can also disrupt normal sex chromosome segregation by interfering with sex chromosome pairing in pachytene, leading to an increase in nondisjunction of up to 6.7%, which is ten times higher than in individuals without B chromosomes. Consequently, some males in this population lacked a Y chromosome, providing evidence that the Y chromosome is not involved in sex determination in Neuroptera [26].

The touch-and-go mechanism is connected to the radial arrangement of the metaphase plate. If the X and Y sex chromosome univalents exhibit amphitelic orientation, and bivalents and univalents are evenly distributed across the metaphase plate, their segregation is controlled by a process called the interaction between spindle fibers that occurs during anaphase I [25]. When anaphase I begins, the univalents lag between the two anaphase groups. Later, in telophase I, the univalents move towards opposite telophase groups. The segregation of X and Y chromosomes using this mechanism has been observed in *Altica* spp. [25,27], *Alagoasa* and *Omophoita* spp. [28] (Coleoptera), Tipulidae (Diptera) [29,30,31,32,33], and in the segregation of X and B chromosomes in *Psylla foersteri* (Homoptera, Psylloidea) [34].

The mechanisms behind touch-and-go and distance pairing, as well as the segregation process based on the interaction of spindle fibers, are present in normal cell structures and their function during meiosis [15,22,35]. Univalent chromosomes have evolved to utilize these structures during their meiotic behavior, depending on the characteristics of univalents. Univalents may attach to both spindle poles (sister centromeres or sister chromatids in holocentric chromosomes), showing amphitelic orientation, or they may attach to only one spindle pole, showing syntelic orientation. If metaphase is radial and univalents are oriented amphitelically, they will exhibit touch-and-go pairing at metaphase I, but if metaphase is non-radial, they will not segregate until anaphase I, where they lag behind and move toward opposite poles in telophase. If univalents are oriented syntelically, they will always move near opposite poles when metaphase I matures and show distance pairing. In all cases, the key factor is pairing at pachytene, which determines the attachment of chromosomes to spindle poles at the very beginning of prometaphase.

The observations in this study strongly support morphology-based [1] and molecular phylogenies [2,3,4,5,6], which place the Sisyridae in a basal position within Neuroptera. Additional cytological research on other sisyrid species, as well as on the families Nevrorthidae (dragon lacewings) and Osmylidae (lance lacewings), is needed before any conclusions can be drawn about the behavior of sex chromosomes in these groups. If the segregation of sex chromosomes also relies on the touch-and-go pairing mechanism in these groups, it would reinforce the idea of the monophyly of Osmyloidea and its taxonomic position relative to other Neuroptera.

Broad et al. [18] and McCulloch and Crowley [19] presented genome assemblies from single female individuals of *S. nigra* and *S. terminalis*, respectively, and scaffolded these assemblies into seven chromosomal pseudomolecules, including the X pseudochromosome. In this study, we determined the diploid chromosome number of male *S. nigra* to be 2*n* = 12 + XY. Therefore, we can infer that NGS-based genome assemblies seem to be a reliable method for determining the haploid chromosome number, particularly for autosomes and the X chromosome. However, it is highly questionable whether there are any criteria for assembling a Y pseudochromosome. In any case, cytological analysis is necessary to provide more precise information about the centromere type and meiotic behavior of chromosomes in species where chromosome number has been inferred from NGS assemblies. In conclusion, to gain a comprehensive and reliable understanding of evolutionary history and the relationships between and within taxa, it is essential to combine data from different observational levels, including morphological, anatomical, cytological, and molecular approaches.

## Data Availability

The original contributions presented in this study are included in the article. Further inquiries can be directed to the corresponding author.
